# The impact of simultaneous batch turn downs and targeted kidney utilization decisions on patient survival

**DOI:** 10.1371/journal.pone.0333222

**Published:** 2026-02-03

**Authors:** Diwakar Gupta, Jingyao Huang, Paola Martin

**Affiliations:** 1 McCombs School of Business, University of Texas, Austin, Texas, United States of America; 2 Bloch School of Business, University of Missouri-Kansas City, Kansas City, Missouri, United States of America; 3 Kelley School of Business, Indiana University, Bloomington, Indiana, United States of America; Hospital Universitario La Paz, SPAIN

## Abstract

In the US, Organ Procurement Organizations (OPOs) procure deceased-donor kidneys, whereas Transplant Programs (TxPs) make utilization decisions for candidates listed at their centers. For each donor, candidates are ranked in a strict priority sequence determined by the national allocation rules. Higher-ranked candidates have the right of first refusal over lower-ranked candidates. TxPs are expected to utilize kidneys based on the merits of performing a transplant for each candidate independently of other candidates. However, they frequently utilize kidneys for lower-ranked candidates. This phenomenon is called list diving. The prevalence of list diving has been documented in the literature but its impact on post-transplant outcomes has not been studied. Moreover, all of the reasons why TxPs exercise list diving are not observed in archival data. Therefore, we examine whether utilization decisions that occur either before or concurrently with multiple declines (referred to as batch turn downs) of higher-ranked candidates result in higher recipient and graft survival after accounting for treatment endogeneity. The out-of-sequence transplants identified via the time-based criterion are referred to as targeted placements (TPs). Such transplants signal TxPs’ exercise of clinical judgment. We find that TPs reduce the waiting time for recipients (average time to transplant: 2.20 years for TP recipients vs. 2.64 years for non-targeted recipients, *p*–value < 0.01) while lengthening it for skipped candidates (average wait time until the first offer: 1.64 years for skipped candidates vs. 1.37 years for non-skipped candidates, *p*–value < 0.01). However, TPs do not significantly improve the survival chances of recipients in aggregate, despite the shortened waiting time. Concurrently, TPs prolong skipped candidates’ waiting time and the extra wait does not improve survival chances of those among them who eventually receive transplants.

## Introduction

In the US, Organ Procurement Organizations (OPOs) procure deceased-donor (DD) organs, Transplant Programs (TxPs) make utilization decisions, and the Organ Procurement and Transplantation Network (OPTN) sets allocation priorities. This paper concerns deceased-donor kidney utilization decisions by TxPs that do not follow the OPTN priority sequence. Because of the acute shortage of kidneys available for transplantation, deviations from the consensus-based priority sequence prescribed by the OPTN result in a perception of unfairness in the national system.

The practice of out-of-sequence utilization by TxPs is called *list diving*. It is commonly practiced and documented in a recent study [1]. While list diving is widespread, the OPTN does not track whether the utilization decisions that do not follow the OPTN priority sequence were clinically necessary or the result of TxPs’ discretion. In this paper, we refer to transplants that were likely the result of TxPs’ discretion as *targeted placement* (abbreviated TP) transplants. With this backdrop, the following questions arise that have not been addressed in the literature.

- How would a researcher identify TP transplants in the historical data?
- What is the impact of TPs on recipients of TP kidneys and on candidates who are skipped in terms of survival?
- What are the implications of TPs for the organ transplantation community?

We use several abbreviations in this paper. For the convenience of the readers, a list of abbreviations is provided in Table 6 in S1 Appendix. A comprehensive review of biomedical, economics, and operations management literature can be found in [2].

The need for this study arises because it is difficult to separate clinically necessary from discretionary deviations. For example, TxPs may choose to skip higher-ranked candidates in the match run on account of kidney size (common in instances involving pediatric donors or recipients), and dual or multi organ transplants. Even after excluding such cases, there may remain specific reasons why skipping some candidates would have been necessary. TxPs have detailed information about candidates’ preferences and health statuses and they have the prerogative to exercise discretion. The OPTN data do not contain sufficient information to separate clinically necessary from discretionary skips, which makes it difficult to study the impact of discretionary skips on outcomes.

To overcome this difficulty, we analyze utilization decisions from a time-based perspective, identifying instances that are not only out-of-sequence but also highly likely to be specifically targeted toward certain candidates based on the timing of the decision. These are instances in which such offer acceptance decisions occur either at the same time or before simultaneous declines of multiple offers. The latter are referred to as batch turn downs. By utilizing a time-based criterion coupled with batch turn downs, our identification strategy learns which deviations are highly-likely to have been discretionary. We utilize a time-based perspective because the timestamp of TxPs’ responses is a key observable variable in OPTN’s match-run data, capturing the progression of TxPs accept/decline decisions.

The Final Rule [3], which is based on the National Organ Transplant Act of 1984 (Pub L. 98-507, 98 Stat. 2339-2348, Oct. 19, 1984), gives candidates and TxPs the right to turn down offers without any negative consequences for access to organs in the future. The intent of this system is to give higher-ranked candidates the right of first refusal while respecting center-level autonomy to exercise clinical judgment. We hypothesize that programs weigh the trade-off between the benefit to a PTR of receiving a kidney at the time of offer versus waiting for a potentially better kidney and increased surgery risk due to deteriorating health. Targeted placements occur when clinical judgment leads TxPs to conclude that the higher-ranked PTRs who are skipped will be better off waiting, while the targeted recipients will be better off receiving a kidney immediately, although both would have been suitable candidates for the offered kidney. The analysis presented in this paper tests this hypothesis.

The contribution of this paper is threefold. (1) We develop a procedure for identifying targeted placements after methodically eliminating all justifiable out-of-sequence placements—e.g., instances involving pediatric donors or recipients, dual transplants, multiorgan transplants, and transplants following bypass actions and open offers by the OPOs. (2) We customize techniques proposed in the literature for the analysis of observational data and fit several models to tease out the impact of TPs on targeted and skipped candidates while accounting for endogeneity. (3) By shining light on the TP phenomenon, we expose a facet of the national organ procurement and utilization system that deserves greater attention by the transplantation community.

## Methods

The following statement is mandated by the OPTN as part of the Data Use Agreement required for access to the national data. “This study used data from the Organ Procurement and Transplantation Network (OPTN). The OPTN data system includes data on all donor, wait-listed candidates, and transplant recipients in the US, submitted by the members of the Organ Procurement and Transplantation Network (OPTN). The Health Resources and Services Administration (HRSA), U.S. Department of Health and Human Services provides oversight to the activities of the OPTN contractor.”

### Data preparation

**Fig 1:**
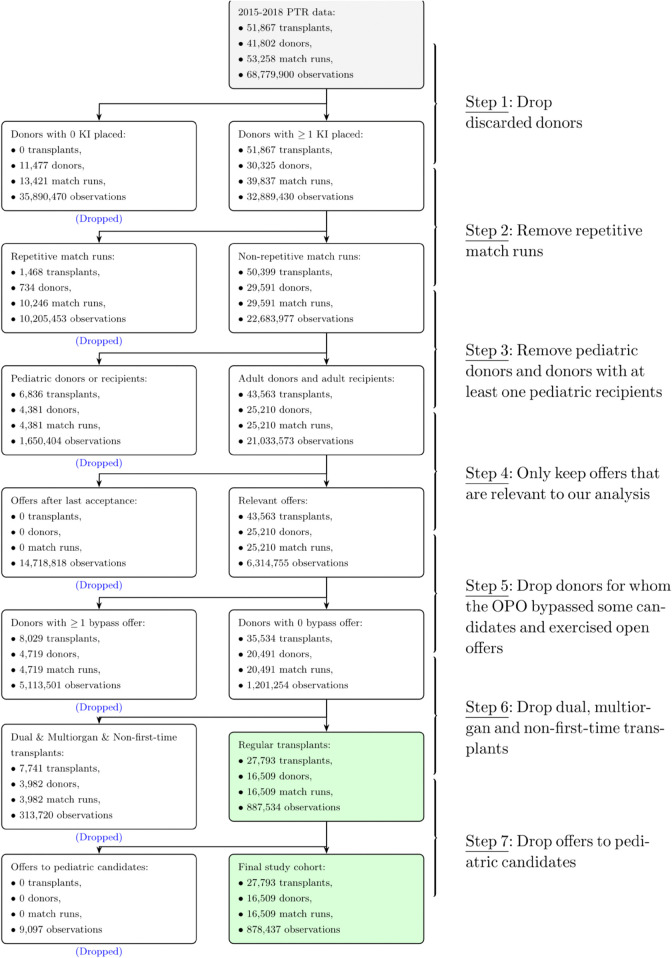
Data Preparation Steps (KI = kidney).

We used the Standard Transplant Analysis and Research (STAR) file data and PTR (match-run) data for the period January 1, 2015 to December 31, 2018, both of which were obtained from UNOS. The match run date is up to December 31, 2018, while the transplant date can be up to January 3, 2019 because there could be some time elapsed from the initiation of the match run to transplant surgery. We also obtained 1-year post-transplant outcomes from the STAR file data up to January 3, 2020. The study period (2015-2018) was selected because the OPTN kidney allocation system [4] remained stable during these years, and the timeframe predated the impact of COVID-19. This study was conducted after obtaining appropriate Institutional Review Board approval. The STAR file contains data on donors, candidates, transplants, and follow-ups (see details in [5]). All IDs are encrypted. The match run (PTR data) contains IDs of donors, candidates and OPOs, along with actual responses, and refusal reasons. Programs may respond with either a “Y” (Accept), “N ”(Decline), or “Z” (Provisional Yes). The latter means that the program would like to keep its option open until it must respond with a final decision. In many cases, a “Z" in our data is not updated even though the kidney is accepted and transplanted. In such instances, we interpret the “Z" as being equivalent to a “Y". Additionally, in some instances, OPOs exercise the bypass option to expedite placements. Typically, this happens when without bypass action, a kidney would become stale, i.e., the accrued Cold Ischemia Time (CIT) would exceed some threshold and the kidney would go unutilized. The expedited placement practice and its impact has been documented in the literature [6–9]. Bypass offers are identified with a “B" in our data either in the initial or the final response data field. In addition, the match-run data contains three date-time variables: the notify date and time, the initial response date and time, and the final response date and time. Of these, only initial and final response date and time are consistently recorded, whereas the notify date and time stamps are missing in 67.2% of instances. Data preparation was carried out in seven steps, which are described in S2 Appendix and presented in Fig 1 as a flow chart.

The purpose of these steps is to eliminate atypical A/D instances as well as instances in which TxPs may justifiably utilize the offered kidney for an out-of-sequence candidate. An observation in our data is a donor-PTR specific kidney match for which the TxP responds with an initial response for the matched PTR listed on the national waitlist. Observations are realized after OPOs perform the match run and make donor data available to TxPs. After completing the seven steps, our study cohort consisted of 27,793 transplants, 16,509 donors, 16,509 match runs, and 878,437 observations.

In the match-run data, TxPs sometimes decline offers for specific reasons, using codes such as 801 (“Patient ill, unavailable, refused, or temporarily unsuitable"). The refusal codes that were in effect at the time when our data were recorded, and their meaning were provided by the OPTN along with the PTR data. Because refusal codes were changed in 2021 [10], we provide a list of most frequently used refusal codes in our data in Table 7 in S3 Appendix. The inclusion of cases in which TxPs chose either 801 or provided specific reasons for refusal may affect our findings. Therefore, we performed a robustness check, labeled RC1, where we removed PTRs where the offer was declined for refusal code 801 or a specific free-text reason was provided, and then repeated our primary analysis.

We next describe the rules we used to label a targeted placement.

### Labeling rules

We consider a transplanted kidney to be a targeted placement if: (1) there were at least 5 eligible PTRs at the same TxP in the match run for which the program submitted an initial response, (2) the TxP’s initial decisions, either “N" or “Z", and initial response times for at least 4 higher ranked candidates were the same, and (3) the time of the program’s initial response for the skipped candidates was either the same or later than the time of its initial response for the recipient for which it indicated its intent to utilize the organ. The four or more higher-ranked PTRs (those with lower PTR sequence number in the match run) that were turned down comprised the batch turn-down (BTD) cohort. By choosing a minimum BTD cohort size of 4, we identify out-of-sequence transplants that TxPs judged to be beneficial for targeted candidates with a high probability. Our approach also strikes a compromise between identifying either too few or too many TPs. To further substantiate our findings, we consider two variants of our approach as robustness checks (RC). Specifically, in RC2, we change the minimum BTD threshold to 3 (from 4), and in RC3, we change the threshold to 5 (from 4). The former results in more instances of TPs and latter in fewer instances. Upon completing the identification procedure, the remainder of our methodology in RC2 and RC3 is identical to that described in this section.

Turning to our main analysis, we find that there are different patterns of A/D responses that may signal to the OPO that the TxP wants to target a kidney to a particular PTR. We identified three such patterns, which we coded into TP labeling rules. In all three cases, criteria (1)–(3) described above hold. The rules vary because of the TxP’s initial response. In particular, in Rule 1, the TxP’s initial response is “N” for the higher-ranked PTRs and either “Y” or “Z” for the lower-ranked PTR. In Rule 2, the TxP’s initial response is “Z” for the higher-ranked PTRs and either “Y” or “Z” for the lower-ranked PTR. If it is “Z”, then the initial and final response date and time are the same. In Rule 3, the TxP’s initial response is “Z” for the higher-ranked PTRs and for the lower-ranked PTR. In all cases, the lower-ranked PTR receives the transplant. Additionally, the final response time of the higher-ranked PTRs is after the final response time of the lower-ranked PTR, or it happens to be on the same day or after the transplant occurred. We provide examples of each of these labeling rules in Tables 8–10 in S4 Appendix.

### Descriptive statistics

We define TP donors as those donors who have at least one kidney transplanted as TP, and the rest as non-targeted placement (NTP) donors. Put differently, none of the NTP donors’ transplants are targeted placements. We define TP recipients as those who receive a TP transplant and NTP recipients as those who receive a NTP transplant. We define BTD recipients as those who are part of a BTD at least once prior to accepting an offer and non-BTD recipients as those recipients who are never part of a BTD prior to their transplant. Lastly, we define BTD candidates as those who are part of a BTD at least once in our study period and non-BTD candidates as those who are never part of a BTD. We provide a visualization of the split of donors and PTRs into the above-mentioned categories in the Descriptive statistics subsection of the Results section.

We compare key characteristics of TP and NTP donors, of TP and NTP recipients, and of BTD and non-BTD candidates. When comparing continuous variables, we first assess whether the assumption of normality would be justified using the Skewness-Kurtosis test. If the data are consistent with the assumption of normality, we perform the unpaired *t*–tests with unequal variances and report the corresponding *p*–values. If not, then we use the non-parametric Mann-Whitney U test to determine the significance of the difference between two groups, and report the corresponding *p*– values and *z*– statistic. When comparing categorical variables, we apply the Chi-square tests and report the *p*–values, along with Cramer’s V as a measure of effect size.

### Impact on TP recipients

We use the OPTN’s TxP performance measure of one-year patient and graft survival (binary), which was in force until July 14, 2022 [11], as the primary measure of transplant success. Our goal is to estimate the impact of targeted placements on survival. Therefore, TP is a treatment in this study. However, the donor—candidate pairs that experience targeted placements are neither randomly selected nor based on uniform criteria. Rather, TxPs exercise differential clinical judgment to choose which donor—candidate pairs are best suited for targeted placements. In other words, targeted placement is an endogenous treatment, a deliberate decision by TxPs.

There are two ways to tease out the treatment effect in the presence of possible endogeneity: (1) by modeling TP as an endogenous treatment associated with some clinical factors plus unobserved judgement, and (2) by modeling TP as an endogenous variable and using an Instrumental Variable (IV) approach [12]. Specifically, the resolution of endogeneity in the first case requires a two-step model with endogenous treatment, while in the second case it requires a two-step model with an IV. Because in our setting TxPs deliberately choose TP transplants, it is more appropriate to treat TP as an endogenous treatment. For this reason, our main analysis utilizes a two-step probit regression with TP as an endogenous treatment. However, for the sake of robustness, we also consider the case in which TP is an endogenous variable and propose an IV model. This analysis is referred to as RC4 and the underlying methodology is described after we first present the endogenous treatment model.

A growing body of research has proposed double machine learning (DML) methods to address endogeneity issues [13]. DML is specifically designed to handle high-dimensional data and potentially non-linear relationships. It tackles endogeneity through orthogonalization and sample splitting: orthogonalization isolates the variation in the treatment variable from confounding factors, while sample splitting reduces overfitting and enhances robustness. We also employ the DML model as a supplement to econometric models. The fitted DML model is referred to as RC5, and it is presented later in this section after we discuss the main model and RC4.

As another robustness check, we perform a placebo test to check if a randomly selected set of transplants will have outcomes similar to those of TP transplants. This test is referred to as RC6 and we discuss the methodology after presenting RC5.

**The Endogenous Treatment Model:** We elaborate on the two-step probit model below. Let *TP*_*ij*_ indicate the binary treatment, i.e., whether the *i*–*j* transplant was a TP transplant, and let *Y*_*ij*_ denote the binary survival outcome, i.e., whether recipient *j* and the graft survived ≥ 1 year after receiving a kidney from donor *i*. The model specification is as follows:


Step 1:

$${TP}_{ij}=\left\{ \begin{array}{cc} 1 & \text{if}\;\alpha_{0}+\alpha_{Z}Z_{ij}+\xi_{ij}>0\\ 0 & \text{otherwise} \end{array}\right.$$ (1)

Step 2:

$$Y_{ij}^{*}=\beta_{0}+\beta_{1}{\hat{TP}}_{ij}+\beta_{D}D_{i}+\beta_{R}R_{j}+\beta_{M}M_{ij}+\beta_{T}T_{ij}+\beta_{G}G_{ij}+\epsilon_{ij};\;\;$$

$$Y_{ij}=1[Y_{ij}^{*}>0].$$ (2)

In Step 1, covariates *Z*_*ij*_ are explanatory variables for the TP decisions. Schold and Kriesche [14] showed that “older and frailer transplant candidates benefit from accepting lower quality organs early after ESRD, whereas younger and healthier patients benefit from receiving higher quality organs even with longer dialysis exposure". Knowing this, TxPs may target older candidates to receive higher KDPI kidneys for whom they estimate that the ideal time window for transplantation may expire if they were to wait longer for a higher-quality kidney. In contrast, the younger candidates could afford to wait longer. Therefore, our first covariate is recipient’s age. The second covariate is KDPI. In addition, TxPs may consider the longevity of recipients, which is reflected in the EPTS score. Therefore, we include EPTS as the third covariate.

In Step 2, ${\hat{TP}}_{ij}$ is the estimated TP decision from the model in Step 1 and $Y_{ij}^{*}$ is a latent variable. In addition, we control for donor, recipient, donor-recipient match, time (year and month) and region fixed effects through regressors *D*_*i*_, *R*_*j*_, *M*_*ij*_, *T*_*ij*_ and *G*_*ij*_, respectively. A complete list of variables that were used in the model, along with summary statistics, is provided in Tables 11 and 12, respectively, in S5 Appendix. Lastly, $\alpha_{0}$ and $\beta_{0}$ are scalars, and $\xi_{ij}$ and $\epsilon_{ij}$ are continuous random terms independent of all other regressors.

In the Impact on targeted placement recipients subsection of the Results section, we report both results when TP is assumed to be either an exogenous or an endogenous treatment. The exogenous treatment model uses *TP*_*ij*_ (the actual TP decision) in Eq (2), instead of ${\hat{TP}}_{ij}$. Full implementation details can be found in the section titled Intro 5 – Treatment assignment features in STATA manual [15].

Lastly, data limitations do not allow us to evaluate what would have happened to TP recipients had they not received the targeted placement. Therefore, we conduct an in silico experiment to assess the survival of TP recipients had they waited the same amount as NTP recipients and received a kidney with a similar KDPI. Specifically, we address the following hypothetical question: What if TP recipients did not receive a targeted placement, continued waiting, and eventually received a non-targeted placement as their NTP recipient counterparts? The experiment is conducted in four steps: 1. We fit Eq (3) to the recipients’ data at the time of transplant to estimate the model’s coefficients, where $\gamma_{0}$ is a scalar and $\eta_{ij}$ is a continuous random term. We then use the fitted Eq (3) to estimate the survival probability of TP recipients based on their current KDPI and time to transplant.

$$Y_{ij}^{*}=\gamma_{0}+\gamma_{D}D_{i}+\gamma_{R}R_{j}+\gamma_{M}M_{ij}+\gamma_{T}T_{ij}+\gamma_{G}G_{ij}+\eta_{ij};\;\;Y_{ij}=1[Y_{ij}^{*}>0].$$ (3)

2. For each TP recipient, we identify a set of NTPs matched to the focal TP recipient based on the following three criteria: (i) they belong to the same TxP, (ii) they belong to the same EPTS group, where we divide EPTS into 20 groups based on the 5th percentiles, and (iii) the NTP recipient’s waiting time is longer than that of the focal TP recipient. 3. For each TP recipient, we compute the average KDPI and average waiting time until transplant for their matched NTPs. 4. Using the fitted Eq (3), we estimate the survival probability of TP recipients by substituting the average KDPI and time to transplant from their matched NTPs. This step aims to measure the survival of TP recipients if they were to wait the same amount of time as their NTP counterparts and receive a kidney with the same KDPI as their NTP counterparts. We then compare the two estimated survival probabilities.

**The Instrumental Variable Model (RC4):** We constructed an instrumental variable (IV) as defined below:


$$IV≐\frac{\text{Sum of all transplants performed by a TxP in the previous 3 months}}{\text{Sum of all transplants performed by all TxPs in the same DSA in the previous 3 months}}.$$

IV is calculated on a monthly basis, i.e., it has the same value for all transplants performed by a TxP in the same month. The intuition behind this instrument is that a dominant program within a DSA, as measured by the proportion of DSA-wide transplants it performs, is likely to have a different pattern of utilizing higher KDPI kidneys. TPs could serve as a mechanism by which TxPs could increase transplant counts without affecting their performance in terms of survival. IV does not directly affect survival outcome of a specific match because it is based on historical utilization counts.

**The DML Model (RC5):** Consider the following partially linear regression model [13].

$$Y=\theta_{0}P+g_{0}(X)+U,\;\;E[U|X,P]=0,$$ (4)

$$P=m_{0}(X)+V,\;\;E[V|X]=0,$$ (5)

where *Y* is the outcome variable (i.e., 1-year survival), *P* is the treatment variable of interest (i.e., TP treatment), vector *X* consists of other controls (including donor, recipient, donor-recipient match characteristics, and time fixed effects), and *U* and *V* are disturbances. The first equation is the main equation, and $\theta_{0}$ is the regression coefficient that we would like to infer. The second equation keeps track of confounding, namely the dependence of the treatment variable on controls. In the DML implementation, the dataset is randomly split into two parts: the main sample and an auxiliary sample. The auxiliary sample is used to partial out the effect of *X* from treatment *P* to obtain the orthogonizalied regressor $V=P-m_{0}(X)$. This orthogonalized regressor is then used in a machine learning estimator to infer $\theta_{0}$ using observations from the main sample [13].

The purpose of orthogonalization is to eliminate the regularization bias commonly associated with machine learning methods. To further reduce bias caused by overfitting, K-fold cross-fitting is often employed. In this approach, the dataset is equally divided into K samples. In the first iteration, the first sample serves as the main sample while the remaining K-1 samples are used as the auxiliary set. In the second iteration, the second sample becomes the main sample and the remaining K-1 samples are used as the auxiliary set. This process is repeated, alternating the roles of the main and auxiliary samples, to generate multiple estimates that are then averaged for robustness.

We utilized the ddml model in STATA [16] for DML implementation, which supports five statistical models: the partially linear model, the interactive model (for binary treatments), the partially linear IV model, the high-dimensional IV model, and the interactive IV model (for binary treatments and instruments). In the case where an instrumental variable is introduced, the DML model regresses the treatment variable on the IV using the auxiliary sample. Since our analysis does not include interaction terms and features only one continuous IV in the IV approach, we applied (1) the partially linear model and (2) the partially linear IV model to examine the impact of TP on survival. We used 4-fold cross-fitting, which is a standard practice for sample splitting.

**The Placebo Test (RC6):** We randomly selected the same number of transplants as the count of TP transplants from the study cohort. These are labeled pseudo-TP transplants. Next, we re-run the exogenous and endogenous models of the Impact on TP recipients subsection of the Methods section where the treatment is the pseudo-TP on the study cohort. Since the random selection of certain transplants as pseudo-TPs may vary from one instance of the experiment to another, we repeated this exercise five times by generating five different pseudo-TP samples.

### Impact on BTD candidates and recipients

Understanding the impact of TPs on skipped candidates is challenging because many skipped candidates do not receive a transplant. We address this challenge by adopting a multi-pronged approach. First, we calculate average statistics on time to transplant, and whether the skipped candidate received a better or worse quality kidney. We calculate the proportion of times that candidates who experience batch turn down eventually receive either lower or higher KDPI kidneys relative to the average KDPI of kidneys for which they were skipped. However, this measure alone does not capture the missed opportunity for skipped candidates. Therefore, we also perform an experiment in silico through which we estimate the number of times a skipped candidate would have received at least as much estimated survival benefit from accepting an offer prior to their actual transplant. Our methodology is described in detail in the sequel.

We calculate the proportion of BTD PTRs that eventually receive a transplant compared to non-BTD PTRs. The OPTN’s reporting requirements from TxPs are different for candidates and recipients, with significantly more data required for the latter group. Because of these data limitations, we were not able to estimate the impact of BTD on individuals who do not receive a transplant during the study period in terms of their expected survival if they were to receive a transplant outside the study period.

Therefore, we focus attention on BTD recipients in additional analysis. We first compare descriptive statistics on the time until transplant for BTD and non-BTD recipients. We also compare the average KDPI of offers received by BTD recipients and the KDPI of their actual transplanted kidney. We examine the proportion of times each recipient eventually receives either a better or worse kidney. Next, we estimate the impact of BTD on BTD recipients’ survival. We follow similar steps as those outlined in the Impact on TP recipients subsection of the Methods section. We do not have reasons to believe that the BTD treatment is endogenous because TxPs choose which PTRs to target, rather than which ones to turn down. Therefore, we fit a probit model with the following specification:

$$S_{ij}^{*}=\delta_{0}+\delta_{1}BTD_{j}+\delta_{D}D_{i}+\delta_{R}R_{j}+\delta_{M}M_{ij}+\delta_{T}T_{ij}+\delta_{G}G_{ij}+\zeta_{ij};\;\;S_{ij}=1[S_{ij}^{*}>0].$$ (6)

The regressors *D*_*i*_, *R*_*j*_, *M*_*ij*_, *T*_*ij*_ and *G*_*ij*_ are the same regressors that we used in Eq (2) of the Impact on TP recipients subsection of the Methods section. The variable *BTD*_*j*_ takes value 1 if recipient *j* was ever part of a BTD and 0 otherwise. The variable *S*_*ij*_ denotes the binary survival outcome, i.e., whether BTD recipient *j* and the graft survived ≥ 1 year after receiving a kidney from donor *i*. The error term is denoted by $\zeta_{ij}$, and $\delta_{0}$ is a scalar.

Lastly, we carry out an experiment in silico to determine the potential impact of the targeted placement practice on BTD recipients. This experiment consists of three steps: (1) We fit the model in Eq (6) to BTD recipients’ data at the time of transplant to estimate its coefficients. (2) We use the fitted the model in Eq (6) to estimate the survival probability associated with each earlier offer that was made to a BTD recipient had that offer resulted in a transplant. (3) We count the number of BTD recipients that have at least one offer for which they are skipped and for which their estimated probability of survival is at least as high as that of the offer that is eventually accepted. The experiment estimates the number of recipients that had at least one offer that would have resulted in either equal or greater probability of survival than the offer that was eventually accepted on their behalf by their TxP.

## Results

All of our results are based on the *study cohort* of 27,793 transplanted kidneys. There are 231 TxPs in our data that performed at least one transplant between 2015 and 2018. Out of these, 138 (59.74%) TxPs performed at least one TP. To ensure that our findings in the Impact on targeted placement recipients subsection of the Results section are not affected by the inclusion of data from TxPs that do not perform any TPs, we present the results for the entire data as well as for a subset which only includes transplants performed by TxPs that performed at least one TP. This subset consists of 23,409 transplants. We refer to these transplants as the *subset cohort*. Note that the majority of the transplants in our data are performed by TxPs that perform at least one TP, which indicates that high-volume TxPs tend to perform TPs.

### Labeling of targeted placements

Out of 27,793 transplanted kidneys, 1,968 (7.08%) were labeled as being targeted placements. In particular, 285, 154, and 1,529 targeted placements were labeled as such by Rule 1, 2, and 3, respectively. The fraction of TPs in the subset cohort is 8.41%.

TP transplants occur at higher PTR sequence numbers compared to NTP transplants, which results in a longer accrued CIT. In fact,the average CIT of TP transplants is statistically longer than that of NTP transplants (20 hours for TP, 16.3 hours for NTP, *p*–value < 0.001). We next investigate whether there are differences in TxPs’ tendency to select targeted recipients by hour and day of week (short term) and by month and year (long-term). Details of the trends of TP percentages are provided in S6–S8 Figs in S6 Appendix. The percentage of times that recipients are selected as a result of targeted placement varies significantly across hours of the day (*p*–value  = 0.001), with a higher percentage occurring during the night, compared to the day. We do not find a statistical difference in terms of the day of the week (*p*–value = 0.502). Furthermore, the number of TPs are relatively stable during the time frame of our study. However, because the total number of transplants has increased over the same time period, the fraction of TPs performed has declined (see S8 Fig in S6 Appendix for the yearly and monthly trend of TP percentages). The increase in the number of transplants has been attributed in part to the Opioid crisis, which has resulted in a new pool of younger (lower KDPI) organ donors [17]. As we show in the next section, older (higher KDPI) donors are more likely to be the subject of targeted placements. Thus, the increase in the number of donors has increased the number of NTPs, but not TPs.

Upon removing PTRs for which the offer was declined for code 801 or a specific free-text reason (RC1) and re-applying TP identification (i.e., BTD threshold as 4) to this subset of our data, we labeled 1,263 cases of TP. Upon changing the BTD threshold to 3 (RC2), we labeled 2,446 transplants as TPs. The increase in the number of TPs is as expected. Similarly, when we changed the minimum BTD threshold to 5 (RC3), we labeled slightly fewer transplants as TPs. In particular, 1,647 transplants were identified as TPs.

### Descriptive statistics

**Fig 2:**
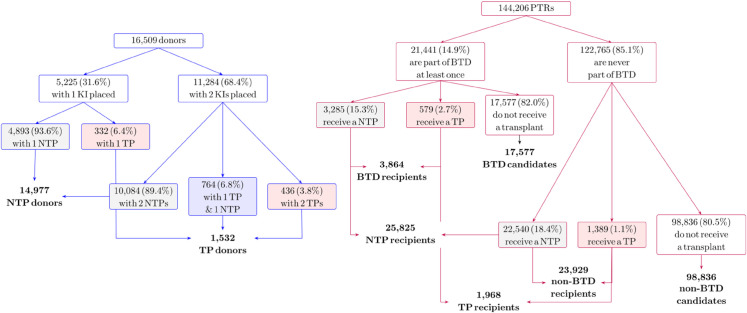
Split of donors and PTRs.

Of the 16,509 donors in our data, 5,225 (31.6%) had one kidney placed, while 11,284 (68.4%) had two kidneys placed. Each transplanted kidney was either a TP or a NTP, resulting in 14,977 NTP donors and 1,532 TP donors. Recall that a donor is classified as a TP donor if at least one kidney is transplanted as a TP. As a result of targeted placement, 21,441 (14.9%) PTRs (out of 144,206 PTRs in the data) were part of a BTD at least once. Among these patients, some received a transplant (targeted or otherwise) during 2015-2018, while others did not. The total number of TP and NTP recipients in our data is 1,968 and 25,825, respectively. Fig 2 provides the split of donors and PTRs.

**Fig 3:**
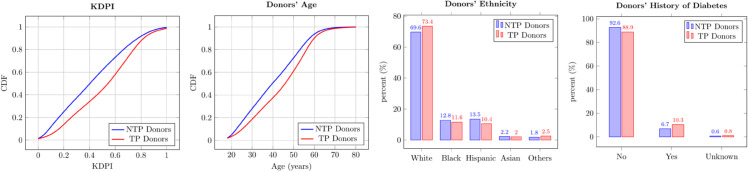
Donor Characteristics Comparison (NTP Donors vs. TP Donors). Average KDPI of NTP and TP donors is 0.42 and 0.51, respectively. Average age of NTP and TP donors is 40.1 and 44.1 years, respectively.

We first compare the key characteristics of TP and NTP donors and present them in Fig 3. The first two panels of Fig 3 show empirical cumulative distribution functions (eCDFs) of KDPI and donors’ age at transplant of TP and NTP donors. We observe that the eCDFs of both KDPI and donors’ age of TPs lie below the corresponding eCDFs of NTPs. This suggests that TP donors are stochastically older and have stochastically higher KDPI compared to NTP donors. For random variables *X* and *Y*, we say that *Y* is stochastically larger than *X* (written $X\leq_{st}Y$) if Ef(X)≤Ef(Y) for all increasing functions f(·) for which the expectations exist. An equivalent condition is P(Y≤x)≤P(X≤x) for all *x* [18]. It is this equivalent condition we use to argue stochastic dominance of TP donors’ age and KDPI. The third and fourth panels of Fig 3 show that TP donors are more likely to be White (*p*–value < 0.01, effect size = 0.03) and a greater proportion of them has diabetes (*p*–value < 0.01, effect size = 0.04). We present a comprehensive comparison of TP and NTP donors’ characteristics along with the test results in Table 13 in S7 Appendix. This table shows that TP donors have higher creatinine and higher protein in urine, are more likely to die from Anoxia and stroke, to have cancer, diabetes, hypertension, myocardial infarction, and to use cigarettes. Donors in the two groups are statistically not different (at 5% level) in terms of gender and blood-borne disease transmission.

**Fig 4:**
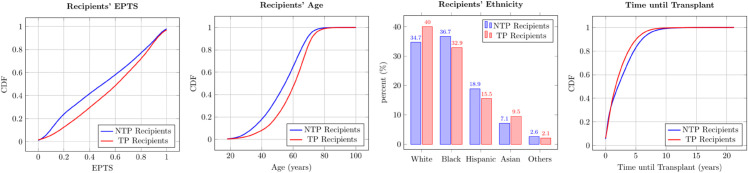
Recipient Characteristics Comparison (NTP Recipients vs. TP Recipients). Average EPTS of NTP and TP recipients is 0.50 and 0.58, respectively. Average age of NTP and TP recipients is 53.1 and 58.7 years, respectively. Average time until transplant of NTP and TP recipients is 2.64 years and 2.19 years, respectively.

Next, we examine the characteristics of TP and NTP recipients. In terms of 1-year survival, a slightly smaller proportion of TP transplants survive (93.90% TP versus 94.88 % NTP, *p*–value  = 0.060, effect size = 0.01). Other key variables are presented in Fig 4 and a more comprehensive comparison can be found in Table 14 in S7 Appendix. The eCDF comparisons in the first, second and the fourth panels suggest that TP recipients have stochastically higher EPTS, are stochastically older and experience stochastically shorter time until transplant. The third panel shows that a greater proportion of TP recipients are White (*p*–value < 0.01, effect size = 0.04).

**Fig 5:**
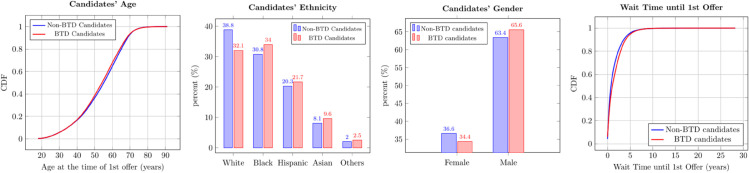
Candidate Characteristics Comparison (Non-BTD Candidates vs. BTD Candidates). Average age at the time of 1st offer for Non-BTD and BTD candidates is 53.3 and 52.7 years, respectively. Average wait time until 1st offer for Non-BTD and BTD candidates is 1.38 years and 1.64 years, respectively.

Lastly, we compare the characteristics of BTD and non-BTD candidates in Fig 5. Note that a candidate may receive multiple offers during 2015-2018. For each candidate, we compute their age and time on waitlist at the time of first offer. As indicated by the eCDF plots in the first and the fourth panels of Fig 5, neither the distribution of age nor the distribution of wait time at first offer of one group stochastically dominates that of the other group, but the mean age at first offer of BTD candidates is significantly lower than that of non-BTD candidates ( Mann-Whitney U results: *p* < 0.01, *z* = −7.20). Additionally, BTD candidates wait longer, on average 1.64 years compared to 1.38 years for non-BTD candidates ( Mann-Whitney U test: *p* < 0.01, *z* = 21.33). BTD candidates are also more likely to be male (*p*–value < 0.01, effect size = 0.02) and Black (*p*–value < 0.01, effect size = 0.05). A comprehensive comparison of candidates in the two groups is presented in Table 15 in S7 Appendix.

### Impact on targeted placement recipients

**The Endogenous Treatment Model:** We report the results of our two-step probit model when TP is considered to be either an exogenous or an endogenous treatment in Table 1 for both cohorts. Table 1 also shows the results of Step 1, which confirms that TxPs are likely to utilize TP decisions when the target recipients are older, have lower EPTS score and the donor’s KDPI is higher.

**Table 1:** Impact of TPs on Survival with Exogenous and Endogenous Treatment Assignment.

	Study Cohort				Subset Cohort			
	Exogenous Treatment		Endogenous Treatment		Exogenous Treatment		Endogenous Treatment	
	Estimate (SE)	ATE	Estimate (SE)	ATE	Estimate (SE)	ATE	Estimate (SE)	ATE
*Step 2:*								
TP	–0.046 (0.050)	–0.005	0.043 (0.615)	0.004	–0.049 (0.051)	–0.005	0.147 (0.670)	0.013
Time FE	Yes	–	Yes	–	Yes	–	Yes	–
Donor FE	Yes	–	Yes	–	Yes	–	Yes	–
Candidate FE	Yes	–	Yes	–	Yes	–	Yes	–
Other FE	Yes	–	Yes	–	Yes	–	Yes	–
Log-likelihood	–5,365.0	–	–12224.9	–	–4,456.6	–	–10,976.0	–
No. of Obs.	27,793	–	27,793	–	23,409	–	23,409	–
*Step 1:*								
Recipient’s Age	–	–	0.026*** (0.002)	–	–	–	0.027*** (0.002)	–
KDPI	–	–	0.395*** (0.051)	–	–	–	0.382*** (0.053)	–
EPTS	–	–	–0.613*** (0.068)	–	–	–	–0.611*** (0.070)	–
*ρ*(e.TP, e.survival)			–0.045				–0.102	

*Note: SE = standard error, ATE = average treatment effect, ρ= Corr, ***p < 0.01, **p < 0.05, *p < 0.1.*

An important finding of our analysis reported in Table 1 is that TP does not significantly affect survival in either the study cohort or the subset cohort, regardless of whether TP is assumed to be an exogenous or endogenous treatment. Put differently, the survival outcomes of TP recipients are not statistically better or worse than those of NTP recipients. We also tested several alternate models in Step 1. In those tests, the size of $\beta_{1}$, the coefficient of ${\hat{TP}}_{ij}$ in Eq (2), varied but remained statistically equal to zero in all cases. Additionally, the correlation among error terms, denoted by *ρ*(e.TP, e.survival) in Table 1, was not significantly different from zero, further confirming that our model adequately captures the effect of TP on survival. Although not intuitive at first glance, the finding that TP treatment does not affect survival is not surprising because TxPs are evaluated based on survival, and they would target specific PTRs only when this action allows them to utilize a kidney from a donor with relatively high KDPI with outcomes similar to those of NTP recipients. Moreover, TxPs that exercise TPs may have different processes in place for post-transplant care to manage survival risk. Our finding that TP does not significantly affect survival is robust even when stratifying the data by ranges of KDPI and EPTS in intervals of 0.2. Such analysis is omitted for brevity.

Upon conducting the in silico counterfactual experiment described in the Impact on TP recipients subsection of the Methods section, we found no significant difference (*p*–value  = 0.9723) between the current survival probability of TP recipients (mean: 93.9%) and their counterfactual survival probability (mean: 93.9%) based on the KDPI and time to transplant of their NTP counterparts. That is, the difference in survival of TP recipients and matched NTP recipients is also insignificant.

**The Instrumental Variable Model (RC4):** We find that IV significantly impacts TP (*p*–values ≤0.01 for both study cohort and subset cohort), but it does not significantly affect survival (*p*–values = 0.381 for study cohort and 0.619 for subset cohort). Therefore, it meets the relevance and exclusion criteria needed to be a viable IV [12]. We present the results of the two-step model with IV in Table 2. Because the IV is defined using the previous 3 months of data, we drop observations that occurred in the first three months of data (January to March 2015). In addition, we drop any observations for which we do not have DSA information. This results in a study cohort of 24,392 transplants and a subset cohort of 20,606 transplants. Table 2 shows that the impact of TP on survival is consistent with our finding in Table 1. That is, TP does not significantly affect survival both when TP is considered an endogenous variable and when it is considered an endogenous treatment.

**Table 2:** Impact of TPs on Survival with IVs.

	Study Cohort		Subset Cohort	
	Estimate (SE)	ATE	Estimate (SE)	ATE
*Step 2:*				
TP	–0.039 (0.363)	–0.004	–0.287 (0.434)	–0.034
Time FE	Yes	–	Yes	–
Donor FE	Yes	–	Yes	–
Candidate FE	Yes	–	Yes	–
Other FE	Yes	–	Yes	–
Log-likelihood	–10,650.6	–	–9,653.3	–
No. of Obs.	24,392	–	20,606	–
*Step 1:*				
*IV*	0.315*** (0.016)	–	0.270*** (0.016)	–
*ρ*(e.TP, e.survival)	–0.006	–	0.113	–

Note: SE = standard error, ATE = average treatment effect, ***p < 0.01, **p < 0.05, *p < 0.1.

**The DML Model (RC5):** Recall from the Methods section that we applied (1) the partially linear model and (2) the partially linear IV model to examine the impact of TP on survival and that we 4-fold cross-fitting. The results of the DML analysis are presented in Table 3. In both models, the coefficient of TP on survival was not statistically significant in either the study cohort or the subset cohort, indicating that TP does not have a significant impact on survival in both cohorts.

**Table 3:** Impact of TPs on Survival under DML Models.

	Study Cohort		Subset Cohort	
	Estimate (SE)	ATE	Estimate (SE)	ATE
*Partial Linear Model:*				
TP	–0.007 (0.006)	–0.007	–0.008 (0.006)	–0.008
Time FE	Yes	–	Yes	–
Donor FE	Yes	–	Yes	–
Candidate FE	Yes	–	Yes	–
Other FE	Yes	–	Yes	–
No. of Obs.	27,793	–	23,409	–
*Partially Linear IV Model:*				
TP	–0.020 (0.040)	–0.020	–0.057 (0.047)	–0.057
Time FE	Yes	–	Yes	–
Donor FE	Yes	–	Yes	–
Candidate FE	Yes	–	Yes	–
Other FE	Yes	–	Yes	–
No. of Obs.	24,392	–	20,606	–

Note: SE = standard error, ATE = average treatment effect, ***p < 0.01, **p < 0.05, *p < 0.1.

**The Placebo Test (RC6):** Upon randomly labeling 1,968 transplants as being TP (labeled as pseudo-TP), and re-running the exogenous and endogenous models of the Impact on TP recipients subsection of the Methods section where the treatment is the pseudo-TP on the study cohort, we obtained the results in Table 4. Note that the analysis is performed only for the study cohort because the subset cohort is defined based on the TP definition. We find that the pseudo-TP does not significantly impact survival. Moreover, the key explanatory variables of recipient’s age, KDPI and EPTS do not statistically impact the pseudo-TP decision. Because the pseudo-TP assignment is based on randomly labeling 1,968 transplants, it could be repeated with different set of transplants labeled as pseudo-TP each time. Upon repeating the experiment with five different random assignments, we consistently find that the pseudo-TP does not significantly impact survival.

**Table 4:** Impact of TPs on Survival under Placebo Test.

	Study Cohort			
	Exogenous Treatment		Endogenous Treatment	
	Estimate (SE)	ATE	Estimate (SE)	ATE
*Step 2:*				
pseudo-TP	–0.019 (0.050)	–0.002	0.139 (2.525)	0.013
Time FE	Yes	–	Yes	–
Donor FE	Yes	–	Yes	–
Candidate FE	Yes	–	Yes	–
Other FE	Yes	–	Yes	–
Log-likelihood	–5,365.3	–		–
No. of Obs.	27,793	–	27,793	–
*Step 1:*				
Recipient’s Age	–	–	0.000 (0.002)	–
KDPI	–	–	0.016 (0.054)	–
EPTS	–	–	0.027 (0.068)	–
*ρ*(e.pseudo-TP, e.survival)			–0.078	

*Note: SE = standard error, ATE = average treatment effect, ρ= Corr, ***p < 0.01, **p < 0.05, *p < 0.1.*

**RC1, RC2 and RC3:** Since RC1, RC2 and RC3 change the count of BTDs in the determination of which transplants are labeled as TPs, we present the results of these robustness checks together. The results are presented in Table 5. This table shows that TP transplants do not exhibit a significantly different 1-year patient and graft survival in any of the three robustness checks, further strengthening our conclusion.

**Table 5:** Results from robustness checks.

RC1 - Removal of 801 refusal code								
	**Study Cohort**				**Subset Cohort**			
	**Exogenous Treatment**		**Endogenous Treatment**		**Exogenous Treatment**		**Endogenous Treatment**	
	**Estimate (SE)**	**ATE**	**Estimate (SE)**	**ATE**	**Estimate (SE)**	**ATE**	**Estimate (SE)**	**ATE**
*Step 2:*								
TP	-0.056 (0.061)	-0.006	-0.473 (0.452)	–0.066	–0.062 (0.062)	–0.006	–0.452 (0.491)	–0.061
Time FE	Yes	–	Yes	–	Yes	–	Yes	–
Donor FE	Yes	–	Yes	–	Yes	–	Yes	–
Candidate FE	Yes	–	Yes	–	Yes	–	Yes	–
Other FE	Yes	–	Yes	–	Yes	–	Yes	–
Log-likelihood	–5,365.0	–	–10,188.6	–	–4,091.0	—	–8,610.3	–
No. of Obs.	27,793	–	27,793	–	21,574	–	21,574	–
*Step 1:*								
Recipient’s Age	–	–	0.036*** (0.002)	–	–	–	0.037*** (0.002)	–
KDPI	–	–	0.481*** (0.060)	–	–	–	0.449*** (0.063)	–
EPTS	–	–	–0.821*** (0.079)	–	–	–	–0.799*** (0.083)	–
*ρ*(e.TP, e.survival)			0.191				0.184	
**RC2 - Batch size of 4**								
	**Study Cohort**				**Subset Cohort**			
	**Exogenous Treatment**		**Endogenous Treatment**		**Exogenous Treatment**		**Endogenous Treatment**	
	**Estimate (SE)**	**ATE**	**Estimate (SE)**	**ATE**	**Estimate (SE)**	**ATE**	**Estimate (SE)**	**ATE**
*Step 2:*								
TP	–0.032 (0.046)	–0.003	–0.040 (0.638)	–0.004	–0.038 (0.047)	–0.004	0.084 (0.754)	0.008
Time FE	Yes	–	Yes	–	Yes	–	Yes	–
Donor FE	Yes	–	Yes	–	Yes	–	Yes	–
Candidate FE	Yes	–	Yes	–	Yes	–	Yes	–
Other FE	Yes	–	Yes	–	Yes	–	Yes	–
Log-likelihood	–5,365.2	–	–13,415.1	–	–4,713.3	–	–12,488.3	–
No. of Obs.	27,793	–	27,793	–	24,862	–	24,862	–
*Step 1:*								
Recipient’s Age	–	–	0.022*** (0.002)	–	–	–	0.022*** (0.002)	–
KDPI	–	–	0.396*** (0.048)	–	–	–	0.391*** (0.049)	–
EPTS	–	–	–0.515*** (0.063)	–	–	–	–0.498*** (0.065)	–
*ρ*(e.TP, e.survival)			0.004				-0.065	
**RC3 - Batch size of 6**								
	**Study Cohort**				**Subset Cohort**			
	**Exogenous Treatment**		**Endogenous Treatment**		**Exogenous Treatment**		**Endogenous Treatment**	
	**Estimate (SE)**	**ATE**	**Estimate (SE)**	**ATE**	**Estimate (SE)**	**ATE**	**Estimate (SE)**	**ATE**
*Step 2:*								
TP	0.015 (0.056)	0.001	–0.162 (0.578)	–0.018	0.007 (0.056)	0.001	–0.038 (0.636)	–0.004
Time FE	Yes	–	Yes	–	Yes	–	Yes	–
Donor FE	Yes	–	Yes	–	Yes	–	Yes	–
Candidate FE	Yes	–	Yes	–	Yes	–	Yes	–
Other FE	Yes	–	Yes	–	Yes	–	Yes	–
Log-likelihood	–5,365.4	–	–11,378.0	–	–4,168.6	–	–9,807.3	–
No. of Obs.	27,793	–	27,793	–	22,145	–	22,145	–
*Step 1:*								
Recipient’s Age	–	–	0.027*** (0.002)	–	–	–	0.028*** (0.002)	–
KDPI	–	–	0.405*** (0.054)	–	–	–	0.392*** (0.057)	–
EPTS	–	–	–0.613*** (0.072)	–	–	–	–0.594*** (0.075)	–
*ρ*(e.TP, e.survival)			0.084				0.023	

*Note: SE = standard error, ATE = average treatment effect, ρ= Corr, ***p < 0.01, **p < 0.05, *p < 0.1*.

### Impact on BTD candidates and recipients

Out of 122,765 non-BTD PTRs, 23,929 (19.5%) received a kidney transplant during our study period. In contrast, out of 21,441 BTD PTRs, 3,864 (18%) received a transplant during our study period. Thus, BTD candidates are slightly less likely to receive a transplant compared to non-BTD candidates.

Focusing on PTRs that eventually received a transplant, we find that the BTD practice leads to statistically longer average time until transplant (3.20 years for BTD recipients vs. 2.51 years for non-BTD recipients, *p*–value < 0.001). However, BTD recipients, on average, receive a better quality kidney. Specifically, among the 3,864 BTD recipients, the average KDPI of kidneys for which they were skipped was 0.57, whereas the average KDPI of transplanted kidneys was 0.44. Looking more closely, we find that 2,443 BTD recipients (63.3%) receive a kidney with better (lower) KDPI, 17 (0.4%) receive a kidney with the same KDPI, 1,404 (36.3%) receive a kidney with worse (higher) KDPI.

From these descriptive statistics, it seems that on the one hand, BTD recipients wait longer to receive a transplant, and on the other hand, the majority receives a higher-quality kidney (lower KDPI). Therefore, the impact of BTD on recipients’ survival is not apparent. We next revert to the model presented in the Impact on BTD candidates and recipients subsection of the Methods section to tease out this impact. We find that the coefficient $\delta_{1}$ in Eq (6) is not significantly different from zero. That is, our model shows that BTD does not lead to statistically different survival after controlling for the covariates mentioned in Eq (6). We report the results of fitting Eq (6) to both data cohorts in Table 16 in S8 Appendix.

We have so far shown that BTD recipients do not have statistically different survival than non-BTD recipients, although they wait longer to receive a transplant. On an intuitive level, this seems to suggest that the lower average KDPI of kidneys that BTD recipients eventually receive compensates for the higher average time on dialysis. Still, some BTD recipients may experience missed opportunities and therefore longer time on dialysis without a net improvement in estimated survival. We calculate the frequency with which this happens from the experiment outlined in the Impact on BTD candidates and recipients subsection of the Methods section. We estimate whether some BTD recipients would have had either the same or better estimated survival had they accepted an offer for which they were skipped. Upon performing the experiment in silico, we find that out of the 3,864 BTD recipients, 3,845 (99.5%) had at least one offer with the same or higher estimated survival probability compared to the offer that their TxP eventually accepted on their behalf, as estimated by Eq (6). In addition, the earliest such offer occurred on average 8.4 months before the actual transplant. To put this in perspective, nearly all BTD recipients could have saved 8.4 months of dialysis time on average and realized similar survival benefit as that from waiting for a lower KDPI kidney had their TxP not practiced TP for earlier offers received on their behalf in our data. Upon omitting offers with refusal code 801 or “other” (the most common refusal codes are explained in Table 7 in S3 Appendix). We find that the number of BTD recipients who had at least one offer with similar survival probability as the one they accepted is 3,836 (99.3%). On average, such offers occurred 8.3 months before the actual offer that was accepted on behalf of these recipients.

Lastly, in RC1, RC2 and RC3, we estimated the number of BTD recipients who would have benefited from an offer for which they were skipped and had higher priority than the individual who was targeted by running the experiment outlined in the Impact on BTD candidates and recipients subsection of the Methods section. We find that these numbers are 2,753 (99.5%), 4,072 (99.4%), and 3,608 (99.5%), for RC1, RC2, and RC3, respectively as compared to 3,845 (99.5%) in the base-case. That is, our findings are robust with respect to slightly different criteria for labeling TPs and the removal of skipped PTRs with a specific refusal reason (either 801 or entered in free text).

## Discussion

This paper tests the authors’ hypothesis that when TxPs make TP decisions, they weigh the trade-off between the benefit to a PTR of receiving a kidney at the time of offer versus waiting for a potentially better kidney and increased transplantation risk due to deteriorating health, and choose TP when in their clinical judgment the skipped PTRs will be better off waiting, while the targeted recipients will be better off receiving a kidney immediately. This hypothesis is tested across all adult kidney transplants during 2015-2028 for which the OPTN data did not have justifiable reasons for skipping higher-ranked candidates.

There are two types of out-of-sequence utilization decisions - those initiated by OPOs and those initiated by TxPs. Both are also referred to as expedited placements. OPO-initiated out-of-sequence placements have been studied in the context of livers [19–21], hearts [22] and kidneys [9,23–25]. The rise in OPO-initiated out-of-sequence placement of kidneys has been attributed to two changes that occurred at about the same time—the circle based allocation rule known as KAS250 [26], and the revised OPO performance metrics [27]. Recent works on this topic include [23] that presents descriptive statistics associated with this practice, and two commentaries [24,25] that raise ethical and fairness concerns about this practice. It is found that out-of-sequence placement and non-use of kidneys available for transplantation have simultaneously increased since the introduction of KAS250 [24]. Neither study evaluates the impact of out-of-sequence offers on outcomes.

Studies related to livers find that the out-of-sequence placement practice varies significantly across OPOs, that it is associated with an increase in utilization of high-risk grafts and an increase in disparities due to race and socioeconomic status. Focusing on heart transplants, [22] shows that out-of-sequence placement of hearts is concentrated in a small subset of OPOs and it is not associated with significantly different 1-year survival. Overall, studies related to all three organs point to a need for further oversight of the out-of-sequence placement practice to ensure equitable and efficient transplantation.

Although the current manuscript is not focused on OPO-initiated actions, its methodology can be adapted to tease out the impact of OPOs decisions on efficiency (e.g., avoidance of non-use), and efficacy (recipients’ survival benefit as well as potential harm to skipped candidates), while separating it from the impact of KAS250 and changes in OPO performance metrics. The literature has not addressed those issues.

List diving is the result of TxPs’ exercise of clinical judgment, which represents the second type of out-of-sequence utilization. This phenomenon has been investigated in [1], but they provide only descriptive statistics. Specifically, they do not estimate the impact of list diving on survival. TP transplants are a subset of the list diving transplants. They constitute instances in which the TxPs signal their preference for utilizing the kidney for a particular recipient based on the timing of their accept/decline decisions and simultaneous batch turn downs. No previous study has considered time-based identification of out-of-sequence decisions.

Our analysis used 1-year patient and graft survival as the primary outcome because it was the metric employed by OPTN to evaluate TxPs’ performance during the study period. However, when choosing the potential recipient, clinicians may additionally consider long-term survival and post-transplant kidney function. These outcomes may play a significant role when 1-year patient and graft survival chances of several potential recipients are statistically indistinguishable, as we observe in our analysis. Therefore, we examine long-term survival differences between TP and NTP transplants. The details of our supplemental analyses are presented in S8 Appendix and our findings are summarized next.

We plot 10-year death-censored Kaplan-Meier (KM) survival curves for TP versus NTP transplants (see S9 Fig in S9 Appendix). For this purpose, we obtained additional survival information up to September 1st 2025, thus the KM survival curves in S9 Fig are also censored by the end of the observation period. The 95% confidence intervals (CI) of the two survival curves overlap, implying that survival chances are not statistically different in aggregate. We present and compare patient and graft survival statistics at specific end points of 1, 3 and 5 years after transplant in Table 17 in S9 Appendix. While 1-year survival is significantly different at 10% level, none of the other comparisons is statistically significant, indicating no model-free differences in long-term survival between TP and NTP transplants. Note that there is no significant difference in 1-year survival upon performing model-based analyses presented earlier in this paper. To test if that finding extends to long-term survival, we re-estimate the coefficients of the endogenous treatment model described in Eq (2) using 3- and 5-year survival outcomes, respectively. The results similarly indicate that TP transplants do not exhibit significantly different 3- or 5-year patient and graft survival relative to NTP transplants (see Tables 18–19 in S9 Appendix).

Turning next to potential differences in post-transplant kidney function between BTD and non-BTD recipients, we estimate the coefficients of Eq (6) using the estimated glomerular filtration rate at 1 year post-transplant (eGFR-1) conditional on 1-year patient and graft survival as the outcome variable. eGFR-1 is a well-established and calculable measure of kidney function based on follow-up data and it has been shown to be positively associated with long-term patient and graft survival [28]. We focus on eGFR measured at 1 year because follow-up data are complete at this time point, whereas data availability is substantially lower at the 3- and 5-year post-transplant horizons. Note that programs are required to report kidney function (i.e., creatinine) at 1 year after transplant. However, this requirement does not extend to 3 and 5 years. The analysis shows that BTD and non-BTD recipients do not significantly differ in kidney function (see Table 20 in S9 Appendix). These supplemental analyses yield insights consistent with our main findings.

Overall, we show that TP transplants do not result in higher survival, either short-term or long-term, relative to non-TP transplants while making the skipped candidates wait longer. Those among the skipped candidates who eventually receive a transplant during the study period, do not experience higher survival benefit or better kidney function. A possible explanation for the lack of significant effect on patient survival due to targeted placements is that centers that practice TP have different processes and operational practices that affect post-transplant survival. Such details are not present in the OPTN data.

Taking a conservative approach to identify TP transplants, we found that during the study period, deviation from the OPTN sequence was not widespread. This contrasts with [1] in which the authors found a much higher (68%) prevalence of list diving within a subset of transplant programs. The difference can be attributed to differences in the study cohort and identification strategy used by [1] and in this study. Still, for the approximately 7% of transplants that could have shortened the waiting time of higher-ranked candidates, the findings of this paper are significant.

Data limitations prevent a comprehensive assessment of what would have happened to TP recipients had they not been targeted. Their potential trajectories, including being removed from waitlist due to deteriorating health, continuing to wait, or receiving a transplant later, cannot be reliably estimated. The in-silico experiment described in the Impact on TP recipients subsection of the Methods section evaluates one specific hypothetical scenario in which TP recipients wait the same amount of time as NTP recipient counterparts and receive a kidney with similar KDPI. We also acknowledge that in some specific cases, e.g., high risk and elderly candidates, the potential harm to such candidates had they not received a TP transplant could have been greater and that clinical judgment autonomy in such cases may explain the occurrence of TPs. The analysis reported in this paper is concerned with the population level effects of TP. We are unable to evaluate individual treatment effects because for any particular transplant only one of two events is observed, i.e., either it is a TP transplant or not. The counterfactual in which the opposite occurs is not observed.

The study also estimates the impact of TP on BTD candidates. It finds that BTD candidates wait longer, do not experience better survival outcomes in instances in which they receive a transplant during the study period, and among the kidneys for which they were skipped, there were many for which they would have received the same survival benefit as they eventually received. In this sense, TP creates a different allocation than that which the OPTN intended.

The study has limitations in terms of its analysis of the impact of TP on BTD candidates. First, turn downs may occur in groups but they may not be followed by a TP transplant. When that happens, certain BTD candidates are not classified as being BTD candidates. Data limitations prevented the authors from identifying such instances. Second, it is possible that some PTRs experienced BTD in years prior to 2015. While it is possible to go back in time to the registration date of each candidate and ascertain whether they experienced a BTD prior to 2015, this exercise presents many challenges. The most significant challenge is that the OPTN allocation policy [4] has changed over time. In particular, it changed in 2014 to a point-based system and then remained stable during 2015-2018. Therefore, the OPTN priority of the same PTR could be different in earlier years, making it difficult to compare BTDs in an apples-to-apples comparison.

The transplantation community needs to reconcile a multitude of considerations when contemplating establishing guidelines concerning the practice of targeted placements. Some such considerations include 1) Ethical considerations (inequity) that arise from not following the OPTN sequence. 2) Need to preserve clinical judgment autonomy since transplant centers have more granular data on PTRs, which are not included in the data collected by the OPTN. 3) Consideration for greater estimated benefit for a subset of TP recipients such as high-risk or elderly populations. By shedding light on this topic, this manuscript highlights the significance of future action on this issue by oversight committees of individual programs and professional societies such as the American Society of Transplantation.

## Disclaimer

The inclusion of the following statement is mandated by the OPTN as part of the Data Use Agreement for access to the national data. “The data reported here have been supplied by UNOS as the contractor for the Organ Procurement and Transplantation Network (OPTN). The interpretation and reporting of these data are the responsibility of the author(s) and in no way should be seen as an official policy of or interpretation by the OPTN or the U.S. Government.”

## Supporting information

S6 Fig Hourly Trend of TP Percentages (All TxPs). (TIFF)

S7 Fig Daily Trend of TP Percentages (All TxPs). (TIFF)

S8 Fig Yearly & Monthly Trend of TP Percentages (All TxPs). (TIFF)

S9 Fig Kaplan-Meier Survival Curves Censored for Death and End of Observation (September 1, 2025). (TIFF)

S1 File S1 Appendix. Table of abbreviations.
(PDF)

S2 File S2 Appendix. Data cleaning steps.
(PDF)

S3 File S3 Appendix. Refusal reasons.
(PDF)

S4 File S4 Appendix. TP labeling rules & examples.
(PDF)

S5 File S5 Appendix. Covariates used in regression models.
(PDF)

S6 File S6 Appendix. Yearly and monthly trend of TP percentage during 2015-2018.
(PDF)

S7 File S7 Appendix. Donor, recipient and candidate characteristics.
(PDF)

S8 File S8 Appendix. Impact of BTD on transplant outcomes.
(PDF)

S9 File S9 Appendix. Additional post-transplant outcomes.
(PDF)
