# Supplementary material for: The impact of simultaneous batch turn downs and targeted kidney utilization decisions on patient survival
Source: PLoS One. 2026 Feb 3;21(2):e0333222. doi: 10.1371/journal.pone.0333222 (PMC12867230; doi:10.1371/journal.pone.0333222)
Supplement: S1 File — Table of abbreviations. (PDF) [file pone.0333222.s005.pdf]

S1 Appendix. Table of abbreviations.

Table 6 provides frequently-used abbreviations.

Table 6. List of frequently-used abbreviations.

A/D	=	Accept/Decline
ATE	=	Average Treatment Effect
BTD	=	Batch Turn-Down
CI	=	Confidence Interval
CIT	=	Cold Ischemia Time
CPRA	=	Calculated Panel Reactive Antibody
DD	=	Deceased Donors
DML	=	Double Machine Learning
DSA	=	Donor Service Area
eCDF	=	Empirical Cumulative Distribution Function
eGFR-1	=	estimated Glomerular Filtration Rate at 1 year post-transplant
EPTS	=	Expected Post Transplant Survival
ESRD	=	End Stage Renal Disease
HLA	=	Human Leukocyte Antigen
HRSA	=	Health Resources and Services Administration
IQR	=	Interquartile Range
IV	=	Instrumental Variable
KDPI	=	Kidney Donor Profile Index
LD	=	Living Donors
LYFT	=	Life Years From Transplant
MCS	=	Mechanical Circulatory Support
NTP	=	Non-targeted Placement
OPO	=	Organ Procurement Organization
OPTN	=	Organ Procurement and Transplantation Network
PTR	=	Potential Transplant Recipient
QALY	=	Quality Adjusted Life Years
RC	=	Robustness Check
STAR	=	Standard Transplant Analysis and Research
TP	=	Targeted Placement
TxP	=	Transplant Program
UNOS	=	United Network for Organ Sharing
