# Supplementary material for: The impact of simultaneous batch turn downs and targeted kidney utilization decisions on patient survival
Source: PLoS One. 2026 Feb 3;21(2):e0333222. doi: 10.1371/journal.pone.0333222 (PMC12867230; doi:10.1371/journal.pone.0333222)
Supplement: S2 File — Data cleaning steps. (PDF) [file pone.0333222.s006.pdf]

S2 Appendix. Data cleaning steps.

Step 1: Of the 41,802 unique potential donors from which at least one kidney was recovered for the purpose of transplantation, we kept the 30,325 donors (32,889,430 observations) who had at least one kidney placed and removed the remaining 11,477.

Step 2: We found 7,983 donors with more than one match run. These can be further divided into two groups: (1) 7,249 donors had at least one match run with incomplete A/D information and one match run with complete information concerning final A/D decisions for all transplanted kidneys; (2) 734 donors had both kidneys placed and the acceptance decision for the two kidneys were found in two different match runs. Multiple match runs usually occur when donor characteristics are updated before acceptance, e.g., cause of death, new lab-test results concerning tissue type, biopsy findings, or donor's infection history [8, Section 5.5]. In the first case, we only kept match runs with complete information on actual transplants found in the STAR file. Incomplete match runs and the corresponding 9,369,262 observations were removed. In the second case, there is no indication in our data as to which donor information were available at which point in time. Therefore, we removed these 734 donors and the corresponding 836,191 observations.

Step 3: The size of a donor's kidney is a critical factor in a TxP's decision to utilize a kidney for a particular PTR. For pediatric donors, such considerations may lead to deviations from typical processes used to evaluate donors. Therefore, we removed pediatric donors (3,098 donors, 1,284,168 observations) as well as adult donors who had at least one pediatric recipient (1,283 donors, 366,236 observations).

Step 4: We removed offers that did not lead to any transplant, since in such cases no targeted placement could occur. Specifically, we removed offers after the first accept if only one kidney was placed (10,445,773 observations) and offers after the second accept if both kidneys were placed (4,273,045 observations).

Step 5: Out of 25,210 unique match runs, 4,719 (18.7%) had at least one bypass decision. We do not consider out-of-sequence utilization decisions that follow bypass actions by the OPOs [22; 27]. We removed these match runs and the corresponding 5,113,501 observations. Open offers may be identified by refusal codes 861 (operational OPO), 862 (donor medical urgency), or 863 (offers not made due to expedited placement attempts). Offers with these refusal codes were not present in our data after

we removed match runs with bypass actions.

Step 6: Out of 35,534 transplants, 234 were dual kidney transplants, 556 were multiorgan transplants, and 4,803 were non-first-time kidney transplants. In a dual transplant, both kidneys from a single donor are transplanted into one recipient. In a multiorgan transplant, at least two organs, one of which is a kidney, from the same donor are transplanted into the same recipient within a short time window. In a non-first-time kidney transplant, the patient had previously received at least one kidney transplant. In all of these cases, either the donors' characteristics, or the recipients' needs are somewhat unique. To avoid confounding our analysis with unknown factors inherent in these uniquely identifiable transplants, we removed all donors with dual transplants and donors whose first kidney is placed in a multi-organ or a non-first-time transplant because the removal of the first transplant influences the PTR sequence number of the second transplant, regardless of whether the second transplant is a regular transplant. For donors whose second kidney is placed through a multiorgan or a non-first-time transplant, we removed the second transplant but kept the first one, which was a regular transplant.

Step 7: We removed offers that were made to pediatric candidates because a TxP's decision to decline such an offer may stem from the fact that the size of the donor's kidney does not match the candidate's need.

After completing these steps, our study cohort consisted of 27,793 transplants, 16,509 donors, 16,509 match runs, and 878,437 observations.
