# Supplementary material for: The impact of simultaneous batch turn downs and targeted kidney utilization decisions on patient survival
Source: PLoS One. 2026 Feb 3;21(2):e0333222. doi: 10.1371/journal.pone.0333222 (PMC12867230; doi:10.1371/journal.pone.0333222)
Supplement: S3 File — Refusal reasons. (PDF) [file pone.0333222.s007.pdf]

S3 Appendix. Refusal reasons.

Table 7 lists the most common refusal reasons, along with their meaning. We provide overall statistics, as well as statistics for refusals that were part of a batch turn down and those that were not. Note that batch turn downs (BTDs) are defined in the Labeling rules section.

Table 7. The comparison in breakdown of top refusal reasons between BTDs versus non-BTDs. Tabulated statistics are number of offers and column percentage. The latter are shown in parentheses.

Top Refusal Reasons and Their Meaning	BTD Offers	Non-BTD Offers	Total
801 (Patient ill, unavailable, refused, or temporarily unsuitable)	17,656 (7.38%)	27,349 (4.28%)	45,005 (5.12%)
830 (Donor age or quality)	155,057 (64.83%)	399,623 (62.51%)	554,680 (63.14%)
836 (Organ anatomical damage or defect)	10,380 (4.34%)	36,415 (5.70%)	46,795 (5.33%)
837 (Organ-specific donor issue)	12,194 (5.10%)	46,109 (7.21%)	58,303 (6.64%)
898 (Other specify)	12,616 (5.28%)	31,887 (4.99%)	44,503 (5.07%)
Other	24,324 (10.17%)	71,044 (11.11%)	95,368 (10.86%)
Missing value	6,935 (2.90%)	26,848 (4.20%)	33,783 (3.85%)
Total number of declined offers	239,162 (100%)	639,275 (100%)	878,437 (100%)

Note: BTD refers to the batch turn downs, which is defined in the Labeling rules section.
