# Supplementary material for: The impact of simultaneous batch turn downs and targeted kidney utilization decisions on patient survival
Source: PLoS One. 2026 Feb 3;21(2):e0333222. doi: 10.1371/journal.pone.0333222 (PMC12867230; doi:10.1371/journal.pone.0333222)
Supplement: S4 File — TP labeling rules & examples. (PDF) [file pone.0333222.s008.pdf]

S4 Appendix. TP labeling rules & examples.

We consider a transplanted kidney to be a targeted placement if: (1) there were at least 5 eligible PTRs at the same TxP in the match run for which the program submitted an initial response, (2) the TxP's initial decisions, either "N" or "Z", and initial response times for at least 4 higher ranked candidates were the same, and (3) the time of the program's initial response for the skipped candidates was either the same or later than the time of its initial response for the recipient for which it indicated its intent to utilize the organ. There are different patterns of A/D responses that may signal to the OPO that the TxP wants to target a kidney to a particular PTR. We identified three such patterns, which we coded into TP labeling rules. In all three cases, criteria (1)–(3) described above hold. The rules vary because of the TxP's initial response.

We provide examples of each of the three TP labeling rules. In all of the examples we present next, we have masked the donor ID and the month in which offers occurred. However, the rest of the data elements shown are as they appeared in the match runs. We specifically chose these examples to highlight that TPs occur at significantly higher PTR sequence numbers than non-targeted placements (NTPs) (average PTR sequence number for TP recipients: 196.6 vs. 30.3 for NTP recipients, $p-$value < 0.01) .

Rule 1: The TxP's initial response is "N" for the higher-ranked PTRs and either "Y" or "Z" for the lower-ranked PTR – see an example in Table 8. All offers shown in the table were made to the same TxP. The initial responses and response times of offers 4–108 are the same as those of offers 2, 3 and 109. We omit these offers for brevity. The TxP indicated its willingness to take a kidney for PTR 110 (highlighted by the box around this offer). This decision was made before the TxP turned down the offers for PTRs 2 through 109. Based on the initial response times, we believe that the TxP targeted PTR 110. That is, PTR 110 received a targeted placement, while PTRs 2 through 109 are part of the batch turn down corresponding to this TP.

Rule 2: The TxP's initial response is "Z" for the higher-ranked PTRs and either "Y" or "Z" for the lower-ranked PTR. If it is "Z", then the initial and final response date and time are the same – see an example in Table 9. We only report offers made to PTRs 9 through 16 for brevity. All of these offers were made to the same TxP.

As shown in Table 9, although the TxP responded with a "Z" for all PTRs, Rule 2 implies a targeted placement because the initial and final response date and time for

Table 8. Example of Labeling Rule 1.

Donor ID	PTR Sequence Number	Initial Response Date, Time	Initial Response	Offer Accept	Targeted Placement
1	1	5/12, 16:14	Z	N	
1	2	5/12, 19:38	N	N	
1	3	5/12, 19:38	N	N	
⋮	⋮	⋮	⋮	⋮	
1	109	5/12, 19:38	N	N	
1	110	5/12, 18:25	Z	Y	Yes

Table 9. Example of Labeling Rule 2.

Donor ID	PTR Sequence Number	Initial Response Date, Time	Initial Response	Offer Accept	Final Response Date, Time	Targeted Placement
2	9	9/20, 0:58	Z	Z	9/20, 0:58	
2	10	9/20, 0:58	Z	Z	9/20, 0:58	
2	11	9/20, 0:58	Z	Z	9/20, 0:58	
2	12	9/20, 0:58	Z	Z	9/20, 0:58	
2	13	9/20, 0:58	Z	Z	9/20, 0:58	
2	14	9/20, 0:58	Z	Z	9/20, 0:58	
2	15	9/20, 0:58	Z	Z	9/20, 0:58	
2	16	9/20, 0:58	Z	Y	9/20, 0:58	Yes

PTR 16 are the same. This means that the TxP signaled right away that it would take the kidney for this PTR. This example also underscores the fact that some TxPs routinely assign a "Z" to all offers and these decisions are often not changed to "N" after the kidney is accepted for another PTR.

Rule 3: The TxP's initial response is "Z" for the higher-ranked PTRs and for the lower-ranked PTR. Additionally, the final response time of the higher-ranked PTRs is after the final response time of the lower-ranked PTR, or it happens to be on the same day or after the transplant occurred – see Table 10 for an example. We only report offers that were made to the same TxP and for PTRs greater than 100. Note that the omitted PTR 103 was listed at a different TxP.

Table 10. Example of Labeling Rule 3.

**Donor ID**	**PTR Sequence Number**	**Initial Response Date, Time**	**Initial Response**	**Offer Accept**	**Final Response Date, Time**	**Targeted Placement**
3	100	10/07, 13:36	Z	N	10/09, 09:53	
3	101	10/07, 13:36	Z	N	10/09, 09:53	
3	102	10/07, 13:36	Z	N	10/09, 09:53	
3	104	10/07, 13:36	Z	N	10/09, 09:53	
3	105	10/07, 13:36	Z	Y	10/09, 06:05	Yes

In Table 10, the initial response of the TxP is "Z" for all PTRs, and the initial and final response times for PTR 105 are not the same. However, the TxP accepted the offer for

this PTR before it made a final decision for all other PTRs included in the batch turn-down.
