# Supplementary material for: The impact of simultaneous batch turn downs and targeted kidney utilization decisions on patient survival
Source: PLoS One. 2026 Feb 3;21(2):e0333222. doi: 10.1371/journal.pone.0333222 (PMC12867230; doi:10.1371/journal.pone.0333222)
Supplement: S5 File — Covariates used in regression models. (PDF) [file pone.0333222.s009.pdf]

S5 Appendix. Covariates used in regression models.

Table 11 shows the covariates used in the regression models. The covariates are presented in the alphabetical order. Table 12 provides summary statistics of these covariates. These statistics are reported for the study cohort of 27,793 transplants.

Table 11. Covariates used in Regression Models.

Donor Covariates	Recipient Covariates	Donor—Recipient Match Covariates	Time Covariates	Region Covariate
Age	Age	HLA mismatches (count)	Month of the tx	Region index
Cause of death	cPRA		Year of the tx	
Creatinine	Dialysis			
Hep C	Diabetes			
History of cancer	EPTS			
History of cigarette use	Functional status			
History of diabetes	Kidney primary diagnosis			
History of hypertension	Insurance type			
History of myocardial infarction	Previous malignancies			
KDPI	Previous pregnancies			
Protein in urine	Ethnicity			
Ethnicity	Time on dialysis			
Risk factors for blood-borne diseases	Weight			

Table 12. Summary Statistics of Covariates Used in Regression Models. The second column shows reported statistics and the third column contains their corresponding values.

Covariates	Reported Statistics	Reported Values
Donors' Age (years)	Mean (Std), IQR	40.46 (13.25), [29-51]
Donors' Cause of Death	% Anoxia	41.30
	% Stroke	25.91
	% Head Trauma	29.93
	% CNS Tumor	0.32
	% Other	2.54
Donors' Terminal Lab Creatinine	Mean (Std), IQR	1.27 (1.13), [0.7-1.4]
Donors' Hep C	% Positive	4.86
Donors' History of Cancer	% No, % Yes, % Unk	97.04, 2.30, 0.67
Donors' History of Cigarette Use	% No, % Yes, % Unk	77.69, 20.50, 1.81
Donors' History of Diabetes	% No, % Yes, % Unk	92.29, 7.07, 0.64
Donors' History of Hypertension	% No, % Yes, % Unk	69.50, 29.69, 0.81
Donors' History of Myocardial infarction	% No, % Yes, % Unk	96.19, 2.93, 0.88
Donors' KDPI	Mean (Std), IQR	0.43 (0.25), [0.21-0.63]
Donors' Protein in Urine	% No, % Yes, % Unk	52.53, 46.96, 0.50
Donors' Ethnicity	% White	69.97
	% Black	12.70
	% Hispanic	13.22
	% Asian	2.19
	% Others	1.91
Donors' Risk Factors for Transmission Blood-Borne Disease	% No, % Yes, % Unk	73.20, 26.79, 0.01
Recipients' Age (years)	Mean (Std), IQR	53.47 (13.03), [45-64]
Recipients' cPRA	Mean (Std), IQR	19.00 (32.42), [0-26]
Recipients' Dialysis	% No, % Yes, % Unk	8.00, 91.81, 0.19
Recipients' Diabetes	% No, % Yes, % Unk	62.26, 37.70, 0.04
Recipients' EPTS	Mean (Std), IQR	0.51 (0.30), [0.22-0.78]
Recipients' Functional Status	% $\leq$ 50%, % $>$ 50%, % Unk	8.6, 88.61, 2.79
Recipients' Kidney Primary Diagnosis	% Type II Diabetes, % Nephrosclerosis, % Other	28.96, 26.54, 44.5
Recipients' Insurance Type	% Private, % Public, % Other	18.18, 81.68, 0.13
Recipients' Previous Malignancies	% No, % Yes, % Unk	91.09, 8.61, 0.30
Recipients' Previous Pregnancies	% No, % Yes, % Unk	91.61, 8.33, 0.06
Recipients' Ethnicity	% White	35.05
	% Black	36.47
	% Hispanic	18.64
	% Asian	7.26
	% Others	2.58
Recipients' Time on Dialysis (years)	Mean (Std), IQR	5.52 (3.18), [3.24-7.2]
Recipients' Weight (Kgs)	Mean (Std), IQR	83.27 (19.25), [69.40-95.82]
Donor-Recipient HLA mismatches	% 0, % 1, % 2, % 3, % 4, % 5, %6	3.59, 0.76, 4.15, 13.47, 28.79, 33.58, 15.60
Region	% 1, % 2, % 3, % 4, % 5, %6	4.07, 12.02, 14.18, 10.61, 15.33, 4.48
	% 7, % 8, % 9, % 10, % 11	7.16, 7.00, 5.80, 8.26, 11.08
Month of transplant	% 1, % 2, % 3, % 4, % 5, %6	8.04, 6.93, 8.60, 7.93, 8.18, 8.12
	% 7, % 8, % 9, % 10, % 11, % 12	8.85, 8.71, 8.57, 8.56, 8.51, 9.00
Year of transplant	% 2015, %2016, % 2017, % 2018, 2019	20.99, 24.06, 26.51, 28.34, 0.10

Note: IQR = Interquartile Range, Unk = Unknown
