# Supplementary material for: The impact of simultaneous batch turn downs and targeted kidney utilization decisions on patient survival
Source: PLoS One. 2026 Feb 3;21(2):e0333222. doi: 10.1371/journal.pone.0333222 (PMC12867230; doi:10.1371/journal.pone.0333222)
Supplement: S6 File — Yearly and monthly trend of TP percentage during 2015-2018. (PDF) [file pone.0333222.s010.pdf]

S6 Appendix. Yearly and monthly trend of TP percentage during 2015-2018.

The overall percentage of TPs is 7.08% during 2015-2018. Figs 6-7 plot the hourly and daily trends of the proportion of TPs among all transplants, respectively. While there are slight differences in the proportion of TPs performed in each hour, there are no differences across days of the week. Fig 8 plots the monthly trend of the proportion of TPs among all transplants for each month from January 2015 to December 2018. A declining trend is attributed to the increase in the number of NTPs due to the Opioid crisis.

S6 Fig 6. Hourly Trend of TP Percentages (All TxPs).

S6 Fig 7. Daily Trend of TP Percentages (All TxPs).

S6 Fig 8. Yearly & Monthly Trend of TP Percentages (All TxPs).
