# Supplementary material for: The impact of simultaneous batch turn downs and targeted kidney utilization decisions on patient survival
Source: PLoS One. 2026 Feb 3;21(2):e0333222. doi: 10.1371/journal.pone.0333222 (PMC12867230; doi:10.1371/journal.pone.0333222)
Supplement: S7 File — Donor, recipient and candidate characteristics. (PDF) [file pone.0333222.s011.pdf]

S7 Appendix. Donor, recipient and candidate characteristics.

Tables 13, 14 and 15 show the comparison of TP and NTP donors, of TP and NTP recipients and of non-BTD and BTD candidates, respectively. For continuous variables, we first assess whether the assumption of normality is justified using the Skewness-Kurtosis test (abbreviated as sktest). If so ($p \geq 0.05$ in sktest for both groups), then we perform unpaired $t-$test assuming unequal variances and report the corresponding $p-$value in tables. If not ($p < 0.05$ in sktest for at least one group), we use the non-parametric Mann-Whitney U test, reporting the $p-$value in tables and using the sign of the $z-$statistics to indicate the direction of group differences. For categorical variables, we apply Chi-square test and report the $p-$values in tables and Cramer's V (i.e., effect size) in the result description below.

Table 13. Summary Statistics of Donor Characteristics. The second column shows the statistics or categories, and the third and fourth columns contain their corresponding values.

Donor Variables	Statistics / Categories	NTP Donors	TP Donors
Age (years)***	Mean (Std), IQR	40.10 (13.21),	44.12 (13.09),
Cause of Death (%)***	Anoxia, Stroke, Head Trauma, CNS Tumor, Other	41.00, 25.51, 30.67, 0.29, 2.52	44.19, 29.90, 22.65, 0.59, 2.68
Terminal Lab Creatinine (mg/dL)***	Mean (Std), IQR	1.23 (1.04),	1.66 (1.72),
Hepatitis C (%)***	Positive	5.06	2.87
History of Cancer (%)***	No, Yes, Unknown	97.20, 2.16, 0.65	95.50, 3.66, 0.85
History of Cigarette Use (%)***	No, Yes, Unknown	78.21, 19.99, 1.80	72.65, 25.46, 1.89
History of Diabetes (%)***	No, Yes, Unknown	92.64, 6.74, 0.63	88.90, 10.31, 0.78
History of Hypertension (%)***	No, Yes, Unknown	70.47, 28.72, 0.80	59.99, 39.10, 0.91
History of Myocardial (%)***	No, Yes, Unknown	96.43, 2.73, 0.84	93.86, 4.83, 1.31
KDPI***	Mean (Std), IQR	0.42 (0.25),	0.51 (0.25),
Protein in Urine (%)***	No, Yes, Unknown	53.13, 46.40, 0.47	46.64, 52.48, 0.85
Ethnicity (%)***	White, Black, Hispanic Asian, Other	69.62, 12.81, 13.51 2.21, 1.85	73.37, 11.62, 10.44 2.02, 2.55
Risk Factors for Blood-Borne Disease Transmission (%)	No, Yes, Unknown	73.10, 26.89, 0.01	74.22, 25.78, 0.00
Gender (%)*	Female, Male	38.53, 61.47	40.73, 59.27

Note: IQR = Interquartile Range. $^{***}p < 0.01$, $^{**}p < 0.05$, $^{*}p < 0.1$

Table 14. Summary Statistics of Recipient Characteristics. The second column shows the statistics or categories, and the third and fourth columns contain their corresponding values.

Recipient Variables	Statistics / Categories	NTP Recipients	TP Recipients
Age (years)***	Mean (Std), IQR	53.07 (13.03), [44-63]	58.71 (11.75), [52-67]
Gender (%)***	Female, Male	39.52, 60.48	34.30, 65.70
CPRA***	Mean (Std), IQR	19.99 (33.14), [0-29]	6.08 (15.81), [0-0]
Dialysis*** (%)	No, Yes, Unknown	7.83, 91.99, 0.18	10.21, 89.53, 0.25
Diabetes (%)***	No, Yes, Unknown	62.72, 37.25, 0.03	56.25, 43.70, 0.05
EPTS***	Mean (Std), IQR	0.50 (0.31), [0.20-0.78]	0.58 (0.28), [0.35-0.82]
Functional Status (%)*	≤ 50%, > 50%, Unknown	8.65, 88.62, 2.73	7.98, 90.04, 1.98
Kidney Primary Diagnosis (%)***	Type II Diabetes<br>Nephrosclerosis<br>Other	28.50<br>26.59<br>44.91	35.01<br>25.97<br>39.02
Insurance Type (%)***	Private, Public, Other	17.91, 81.95, 0.14	21.80, 78.15, 0.05
Previous Malignancies (%)**	No, Yes, Unknown	91.18, 8.51, 0.31	89.94, 9.96, 0.10
Previous Pregnancies ***	% No, % Yes, % Unk	8.46%, 30.96%, 60.58%	6.79%, 27.59%, 65.75%
Ethnicity (%)***	White, Black, Hispanic Asian, Other	34.68, 36.74, 18.88 7.09, 2.61	39.99, 32.88, 15.55 9.45, 2.13
Time on Dialysis (years)***	Mean (Std), IQR	5.63 (3.20), [3.37-7.31]	3.95 (2.30), [2.21-5.32]
Weight (Kg)***	Mean (Std), IQR	83.38 (19.34), [69.40-96.02]	81.83 (18.00), [69.40-93.09]

Note: IQR = Interquartile Range. $^{***}p < 0.01$, $^{**}p < 0.05$, $^{*}p < 0.1$.

Table 15. Summary Statistics of Candidate Characteristics. The second column shows the statistics or categories, and the third and fourth columns contain their corresponding values.

Candidate Variables	Statistics / Categories	non-BTD Candidates	BTD Candidates
Age at time of 1st offer (years)***	Mean (Std), IQR	53.32 (12.87), [44.97, 63.26]	52.70 (12.67), [44.30, 62.35]
Gender (%)***	Female, Male	36.55% , 63.45%	34.37%, 65.63%
Ethnicity (%)***	White, Black, Hispanic Asian, Other	38.78%, 30.85%, 20.27%, 8.11%, 2.00%	32.06%, 34.02%, 21.74%, 9.63%, 2.53%
EPTS***	Mean (Std), IQR	0.56 (0.30), [0.30, 0.84]	0.58 (0.30), [0.30, 0.86]
Weight (Kg)***	Mean (Std), IQR	83.95 (19.80), [69.85, 96.62]	85.04 (20.05), [70.31, 98.3]
Diabetes (%)	No, Yes, Unknown	55.11%, 43.80%, 0.1%	55.08%, 44.80%, 0.11%
Insurance Type (%)	Private, Public, Other	43.64%, 56.07%, 0.29%	43.31%, 56.31%, 0.38%
Waiting time until 1st offer (years)***	Mean (Std), IQR	1.38 (1.70), [0.24, 1.94]	1.65 (1.76), [0.29, 2.48]

Note: IQR = Interquartile Range. $^{***}p < 0.01$, $^{**}p < 0.05$, $^{*}p < 0.1$.

There are three continuous donor variables: age, terminal lab creatinine, and KDPI. Through the sktest, we that none of the variables are normally distributed, i.e., $p < 0.05$ for both TP and NTP donors. Therefore, we apply the Mann-Whitney U test. All three variables show significant differences between the two groups ($p-$values shown in Table 13). Compared to NTP, TP donors are statistically older ($z = 11.37$), have higher KDPI ($z = 14.10$) and higher creatinine ($z = 7.28$). Additionally, We find that donors in the two groups are statistically not different in terms of blood-borne disease transmission (effect size = 0.01) and gender at 5% significance level (effect size = 0.01). TP donors are more likely to die from Anoxia and stroke (effect size =0.05), to have protein in urine (effect size = 0.04), to have cancer (effect size = 0.03), diabetes (effect size = 0.04), hypertension (effect size = 0.07), myocardial infarction (effect size = 0.04), and to use cigarettes (effect size = 0.04). They are less likely to have Hepatitis C (effect size = 0.03).

Regarding recipients, there are five continuous variables: age, CPRA, EPTS, time on dialysis and weight. None of the variables show normality. We use Mann-Whitney U test and find that compared to NTP recipients, TP recipients are older ($z = 19.00$), have statistically shorter time on dialysis ($z = -22.30$), have higher EPTS ($z = 10.54$), lower CPRA ($z = -17.35$) and lower weight ($z = -3.26$). Additionally, TP recipients are less likely to receive dialysis treatment (effect size = 0.02) and have previous pregnancy (effect size = 0.03), more likely to have diabetes (effect size = 0.03), more likely to have previous malignancies (effect size = 0.02), and private insurance (effect size = 0.03). TP recipients are more likely to be White (effect size = 0.04) and males (effect size = 0.03). The two groups of recipients are not different in functional status at 5% significance level (effect size = 0.01).

There are four continuous candidate variables: age at time of 1st offer, EPTS, weight, and wait time until 1st offer. None of them show normality. We apply Mann-Whitney U test and find that compared to non-BTD candidates, BTD candidates have higher EPTS ($z = 5.57$), higher weight ($z = 6.79$), wait longer until the 1st offer ($z = 21.33$) and they have lower age at time of the 1st offer ($z = -7.20$). In addition, BTD candidates are more likely to be males (effect size = 0.02) and non-Whites (effect size = 0.05). The two groups are not different in terms of insurance types (effect size = 0.006) and diabetes status (effect size = 0.002).
