# Supplementary material for: The impact of simultaneous batch turn downs and targeted kidney utilization decisions on patient survival
Source: PLoS One. 2026 Feb 3;21(2):e0333222. doi: 10.1371/journal.pone.0333222 (PMC12867230; doi:10.1371/journal.pone.0333222)
Supplement: S8 File — Impact of BTD on transplant outcomes. (PDF) [file pone.0333222.s012.pdf]

S8 Appendix. Impact of BTD on transplant outcomes.

Table 16 shows the output of Eq (6) and highlights the impact of BTD on 1-year patient and graft survival.

Table 16. Impact of BTD on Survival.

	**Study Cohort**		**Subset Cohort**	
	Estimate (SE)	ATE	Estimate (SE)	ATE
BTD	0.052 (0.039)	0.005	0.050 (0.040)	0.005
Time FE	Yes	–	Yes	–
Donor FE	Yes	–	Yes	–
Candidate FE	Yes	–	Yes	–
Log-likelihood	-5,364.5	–	-4,456.2	–
No. of Obs.	27,793	–	23,409	–

Note: SE =standard error, ATE = average treatment effect, $^{***}p < 0.01$, $^{**}p < 0.05$, $^{*}p < 0.1$.
