# Supplementary material for: The impact of simultaneous batch turn downs and targeted kidney utilization decisions on patient survival
Source: PLoS One. 2026 Feb 3;21(2):e0333222. doi: 10.1371/journal.pone.0333222 (PMC12867230; doi:10.1371/journal.pone.0333222)
Supplement: S9 File — Additional post-transplant outcomes. (PDF) [file pone.0333222.s013.pdf]

S9 Appendix. Additional post-transplant outcomes.

To estimate long-term survival differences between TP and NTP transplants, we first plot 10-year death-censored Kaplan-Meier (KM) survival curves for TP versus NTP transplants (see Fig 9). For this purpose, we obtained additional survival information up to September 1st 2025, thus the KM survival curves in Fig 9 are also censored by the end of the observation period. Fig 9 shows that the two survival curves are not statistically different as the 95% confidence intervals (CI) of the two survival curves overlap. We present and compare patient and graft survival statistics at specific end points of 1, 3 and 5 years after transplant (see Table 17). While 1-year survival is significantly different at 10% level, none of the other comparisons is statistically significant, indicating no model-free differences in long-term survival between TP and NTP transplants. Note that there is no significant difference in 1-year survival upon performing model-based analyses presented earlier in this paper. To test if that finding extends to long-term survival, we re-estimate the coefficients of the endogenous treatment model described in Eq (2) using 3- and 5-year survival outcomes, respectively. The results similarly indicate that TP transplants do not exhibit significantly different 3- or 5-year patient and graft survival relative to NTP transplants (see Tables 18 and 19).

S9 Fig 9. Kaplan-Meier Survival Curves Censored for Death and End of Observation (September 1, 2025)

Table 17. Model-free Comparison of Post-Transplant Survival for TP versus NTP Transplants

	TP Transplants	NTP Transplants	$p-$value
1-year survival	93.90%	94.88%	0.0603
3-year survival	86.64%	87.07%	0.5836
5-year survival	75.41%	76.65%	0.2106

To examine differences in post-transplant kidney function between BTD and non-BTD recipients, we re-estimate the coefficients of Eq (6) using the estimated glomerular filtration rate at 1 year after transplant (eGFR-1) conditional on 1-year patient and graft survival as the outcome variable, which is a well-established and calculable measure of kidney function and has been shown to be positively associated with long-term survival. We focus on eGFR measured at 1 year because follow-up data are complete at this time point, whereas data availability is substantially lower at the 3-

Table 18. Impact of TPs on 3-Year Survival with Exogenous and Endogenous Treatment Assignment.

	Study Cohort				Subset Cohort			
	Exogenous Treatment		Endogenous Treatment		Exogenous Treatment		Endogenous Treatment	
	Estimate (SE)	ATE	Estimate (SE)	ATE	Estimate (SE)	ATE	Estimate (SE)	ATE
Step 2:								
TP	0.006 (0.040)	0.001	-0.425 (0.361)	-0.103	0.008 (0.040)	0.002	-0.342 (0.402)	-0.080
Time FE	Yes	–	Yes	–	Yes	–	Yes	–
Donor FE	Yes	–	Yes	–	Yes	–	Yes	–
Candidate FE	Yes	–	Yes	–	Yes	–	Yes	–
Other FE	Yes	–	Yes	–	Yes	–	Yes	–
Log-likelihood	-10,195.6	–	-17,054.9	–	-8,569.8	–	-15,089.0	–
No. of Obs.	27,793	–	27,793	–	23,409	–	23,409	–
Step 1:								
Recipient's Age	–	–	0.026*** (0.002)	–	–	–	0.027*** (0.002)	–
KDPI	–	–	0.393*** (0.051)	–	–	–	0.381*** (0.053)	–
EPTS	–	–	-0.610*** (0.068)	–	–	–	-0.608*** (0.070)	–
ρ(e.TP, e.survival)			0.207				0.173	

Note: SE = standard error, ATE = average treatment effect, $\rho =$ *Corr,* $^{***}p < 0.01$, $^{**}p < 0.05$, $^{*}p < 0.1$.

Table 19. Impact of TPs on 5-Year Survival with Exogenous and Endogenous Treatment Assignment.

	Study Cohort				Subset Cohort			
	Exogenous Treatment		Endogenous Treatment		Exogenous Treatment		Endogenous Treatment	
	Estimate (SE)	ATE	Estimate (SE)	ATE	Estimate (SE)	ATE	Estimate (SE)	ATE
Step 2:								
TP	-0.002 (0.150)	0.000	$-0.430^{\dagger}$ (0.426)	-0.139	-0.002 (0.035)	0.000	$-0.435^{\dagger}$ (0.433)	-0.141
Time FE	Yes	–	Yes	–	Yes	–	Yes	–
Donor FE	Yes	–	Yes	–	Yes	–	Yes	–
Candidate FE	Yes	–	Yes	–	Yes	–	Yes	–
Other FE	Yes	–	Yes	–	Yes	–	Yes	–
Log-likelihood	-14,328.6	–	-21,316.7	–	-12,050.9	–	-18,693.8	–
No. of Obs.	27,793	–	27,793	–	23,409	–	23,409	–
Step 1:								
KDPI	–	–	0.553*** (0.051)	–	–	–	0.544*** (0.052)	–
EPTS	–	–	0.224*** (0.043)	–	–	–	0.237*** (0.044)	–
ρ(e.TP, e.survival)			0.207				0.215	

Note: SE =standard error, ATE = average treatment effect, $^{***}p < 0.01$, $^{**}p < 0.05$, $^{*}p < 0.1$.

†: We used only KDPI and EPTS as stage-1 covariates in endogenous treatment model for 5-year survival to fully remove the endogeneity.

and 5-year post-transplant horizons. Note that programs are required to report kidney function (i.e., creatinine) at 1 year after transplant. However, this requirement does not extend to 3 and 5 years. The results show that BTD and non-BTD recipients do not significantly differ in kidney function (Table 20). The numbers of observations in this table are smaller than those in other tables because eGFR-1 is calculated conditional on 1-year survival.

Table 20. Impact of BTD on eGFR-1.

	**Study Cohort**		**Subset Cohort**	
	Estimate (SE)	ATE	Estimate (SE)	ATE
BTD	-0.253 (0.355)	-0.253	-0.067 (0.359)	-0.067
Time FE	Yes	–	Yes	–
Donor FE	Yes	–	Yes	–
Candidate FE	Yes	–	Yes	–
Adjusted R-squared	0.2516	–	0.2499	–
No. of Obs.	25,958	–	21,884	–

Note: SE =standard error, ATE = average treatment effect, $^{***}p < 0.01$, $^{**}p < 0.05$, $^{*}p < 0.1$.
